# *In Vitro* Activity of the Siderophore Cephalosporin, Cefiderocol, against Carbapenem-Nonsusceptible and Multidrug-Resistant Isolates of Gram-Negative Bacilli Collected Worldwide in 2014 to 2016

**DOI:** 10.1128/AAC.01968-17

**Published:** 2018-01-25

**Authors:** Meredith A. Hackel, Masakatsu Tsuji, Yoshinori Yamano, Roger Echols, James A. Karlowsky, Daniel F. Sahm

**Affiliations:** a International Health Management Associates, Inc., Schaumburg, Illinois, USA; b Drug Discovery and Disease Research Laboratory, Shionogi & Co., Ltd., Osaka, Japan; c Pharmaceutical Research Division, Shionogi & Co., Ltd., Osaka, Japan; d Clinical Development and Medical Affairs, ID3C, LLC, Easton, Connecticut, USA; e Department of Medical Microbiology, College of Medicine, University of Manitoba, Winnipeg, Manitoba, Canada

**Keywords:** cefiderocol, siderophore, carbapenem-nonsusceptible, multidrug-resistant, Gram-negative bacilli

## Abstract

The *in vitro* activity of the investigational siderophore cephalosporin, cefiderocol (formerly S-649266), was determined against a 2014–2016, 52-country, worldwide collection of clinical isolates of carbapenem-nonsusceptible Enterobacteriaceae (*n* = 1,022), multidrug-resistant (MDR) Acinetobacter baumannii (*n* = 368), MDR Pseudomonas aeruginosa (*n* = 262), Stenotrophomonas maltophilia (*n* = 217), and Burkholderia cepacia (*n* = 4) using the Clinical and Laboratory Standards Institute (CLSI) standard broth microdilution method. Iron-depleted cation-adjusted Mueller-Hinton broth (ID-CAMHB), prepared according to a recently approved (2017), but not yet published, CLSI protocol, was used to test cefiderocol; all other antimicrobial agents were tested using CAMHB. The concentration of cefiderocol inhibiting 90% (MIC_90_) of isolates of carbapenem-nonsusceptible Enterobacteriaceae was 4 μg/ml; cefiderocol MICs ranged from 0.004 to 32 μg/ml, and 97.0% (991/1,022) of isolates demonstrated cefiderocol MICs of ≤4 μg/ml. The MIC_90_s for cefiderocol for MDR A. baumannii, MDR P. aeruginosa, and S. maltophilia were 8, 1, and 0.25 μg/ml, respectively, with 89.7% (330/368), 99.2% (260/262), and 100% (217/217) of isolates demonstrating cefiderocol MICs of ≤4 μg/ml. Cefiderocol MICs for B. cepacia ranged from 0.004 to 8 μg/ml. We conclude that cefiderocol demonstrated potent *in vitro* activity against a 2014–2016, worldwide collection of clinical isolates of carbapenem-nonsusceptible Enterobacteriaceae, MDR A. baumannii, MDR P. aeruginosa, S. maltophilia, and B. cepacia isolates as 96.2% of all (1,801/1,873) isolates tested had cefiderocol MICs of ≤4 μg/ml.

## INTRODUCTION

Carbapenems are broad-spectrum antimicrobial agents that serve as therapies of last resort for many Gram-negative bacterial infections. Regrettably, carbapenem resistance and multidrug resistance have emerged in clinical isolates of Enterobacteriaceae and nonfermentative Gram-negative bacilli, including Pseudomonas aeruginosa, Acinetobacter baumannii, Stenotrophomonas maltophilia, and Burkholderia cepacia, and are of increasing concern in the treatment of patients infected with these pathogens (1–4). The battery of antimicrobial agents currently available to treat patients infected with carbapenem-resistant and multidrug-resistant (MDR) Gram-negative bacilli includes aminoglycosides, tigecycline, ceftazidime-avibactam, meropenem-vaborbactam, ceftolozane-tazobactam, and colistin; however, each of these agents is commonly associated with significant toxicities (aminoglycosides, tigecycline, and colistin), increasing resistance (aminoglycosides and tigecycline), inactivity against one or more classes of β-lactamase enzymes (ceftazidime-avibactam, meropenem-vaborbactam, and ceftolozane-tazobactam), or, in the case of colistin, intrinsic resistance to several species of Enterobacteriaceae (Proteus spp., Providencia spp., Morganella morganii, and Serratia spp.) (5). In the case of the β-lactam/β-lactamase inhibitor combinations, ceftazidime-avibactam is inactive against carbapenem-resistant isolates producing class B metallo-β-lactamases (e.g., NDM, IMP, and VIM); meropenem-vaborbactam is inactive against both class B and OXA-48 (class D) β-lactamases, while ceftolozane-tazobactam is susceptible to hydrolysis by all carbapenemases including class A (e.g., KPC), class B, and class D enzymes as well as by AmpC (class C) β-lactamases (1, 2, 6–9).

New antimicrobial agents are needed to outpace the increasing prevalence of, and diversification in, antimicrobial resistance in Gram-negative bacilli (10). One of the main impediments to the effectiveness of antimicrobial agents against Gram-negative bacteria is hindered transport across the bacterial outer membrane to gain access to their sites of action. For example, porin channels, particularly in P. aeruginosa, are not efficient, and efflux pumps remove many antimicrobial agents that do gain access to the periplasmic space and provide a barrier to antimicrobial agent ingress to their sites of action along the bacterial cell membrane or within the cytoplasm. Cefiderocol, formerly S-649226, is a parenteral siderophore cephalosporin that has a unique mechanism of bacterial cell entry and that is currently in clinical development. The catechol moiety (siderophore) at the three-position side chain of cefiderocol's cephalosporin promotes formation of a chelated complex with ferric iron and facilitates its transport across the outer membrane of Gram-negative bacilli using their receptor-mediated bacterial iron transport systems (11). The cephalosporin moiety of cefiderocol binds primarily to bacterial penicillin binding protein 3 (PBP3) (11, 12). In previous studies, cefiderocol demonstrated *in vitro* activity against carbapenemase-producing Gram-negative bacilli and was reported to be more stable than other β-lactam agents such as ceftazidime, cefepime, and meropenem against class A (KPC), B (VIM, IMP, and NDM), and D (OXA) carbapenemases (13–15). Cefiderocol has also been reported to be active against extended-spectrum β-lactamase (ESBL)-producing Escherichia coli and Klebsiella pneumoniae (13) as well as against meropenem-resistant P. aeruginosa and A. baumannii (16).

The Clinical and Laboratory Standards Institute (CLSI) Subcommittee on Antimicrobial Susceptibility Testing has approved, but not yet published, broth microdilution and disk diffusion methods and quality control MIC ranges for *in vitro* testing of cefiderocol (17–19). *In vitro* susceptibility testing of cefiderocol by broth microdilution requires the use of iron-depleted cation-adjusted Mueller-Hinton broth (ID-CAMHB) to ensure induction of ferric iron transporters and testing conditions that reflect the *in vivo* host environment during infection (13, 15, 17, 20). Cefiderocol MICs determined using ID-CAMHB are reproducible and correlate with *in vivo* efficacy in animal models (21–23). Cefiderocol MICs determined in CAMHB with iron concentrations of >0.03 μg/ml (non-iron-depleted conditions) are variable and do not correlate with *in vivo* efficacy (22).

In the current study, we tested a 2014–2016 collection of 1,873 clinical isolates of Gram-negative bacilli provided by a worldwide network of laboratories (52 countries) against cefiderocol and relevant comparative agents using the current CLSI broth microdilution methodology (5, 24).

## RESULTS

**FIG 1:**
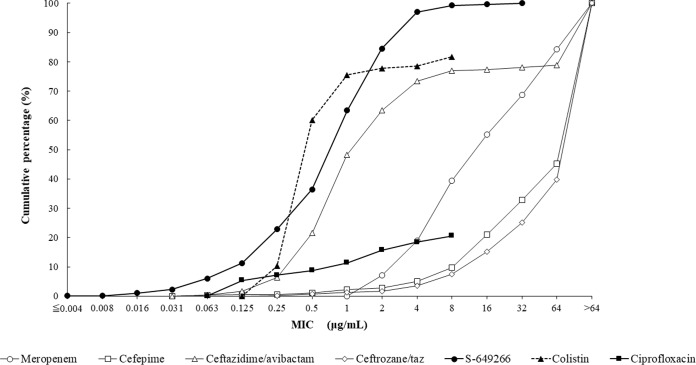
Cumulative cefiderocol MIC distribution (percentage of isolates) for 1,022 isolates of meropenem-nonsusceptible Enterobacteriaceae.

**FIG 2:**
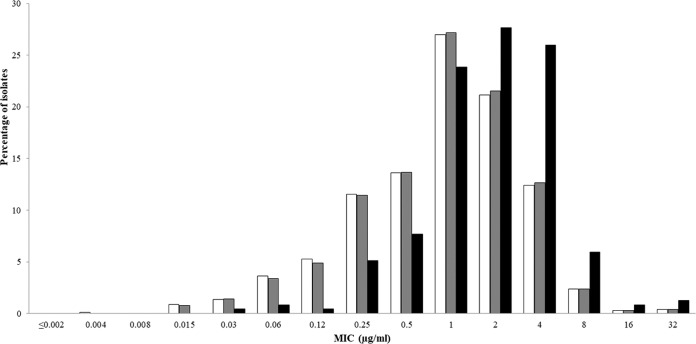
Cefiderocol MIC distributions for all isolates of carbapenem-nonsusceptible Enterobacteriaceae (white bars; *n* = 1,022), isolates of carbapenem-nonsusceptible Enterobacteriaceae that were concurrently nonsusceptible to ceftolozane-tazobactam (gray bars; *n* = 1,005), and isolates of carbapenem-nonsusceptible Enterobacteriaceae that were concurrently nonsusceptible to ceftazidime-avibactam (black bars; *n* = 235).

Cefiderocol at a concentration of 4 μg/ml inhibited 96.2% (1,801/1,873) of all Gram-negative isolates tested in the current study. The *in vitro* activities of cefiderocol and comparative agents against the 1,022 isolates of carbapenem-nonsusceptible Enterobacteriaceae tested are summarized in Table 1. The concentrations of cefiderocol inhibiting 50% (MIC_50_) and 90% (MIC_90_) of isolates of carbapenem-nonsusceptible Enterobacteriaceae were 1 and 4 μg/ml, respectively. The cumulative percentage of isolates of carbapenem-nonsusceptible Enterobacteriaceae inhibited at various MICs of each agent tested is shown in Fig. 1. Enterobacter spp. demonstrated higher MIC_50_ (2 μg/ml) and MIC_90_ (8 μg/ml) values than the other genera/species of Enterobacteriaceae tested. The MIC range for cefiderocol for carbapenem-nonsusceptible Enterobacteriaceae was 0.004 to 32 μg/ml, with 97.0% (991/1,022) of isolates having cefiderocol MICs of ≤4 μg/ml (Fig. 2). The MIC_50_ and MIC_90_ values for isolates with concurrent carbapenem-nonsusceptible and ceftolozane-tazobactam-nonsusceptible phenotypes (*n* = 1,005) were 1 and 4 μg/ml, respectively, and for carbapenem-nonsusceptible and ceftazidime-avibactam-nonsusceptible phenotypes (*n* = 235), they were 2 and 4 μg/ml, respectively (Table 2). Less than 80% of carbapenem-nonsusceptible Enterobacteriaceae were susceptible to either ceftazidime-avibactam (77.0% susceptible; MIC_90_, >64 μg/ml) or colistin (77.8% susceptible; MIC_90_, >8 μg/ml) (Table 1). The cefiderocol MIC distribution demonstrated a rightward shift to slightly higher MICs for isolates of carbapenem-nonsusceptible Enterobacteriaceae concurrently nonsusceptible to ceftazidime-avibactam than for all carbapenem-nonsusceptible isolates and isolates of carbapenem-nonsusceptible isolates concurrently nonsusceptible to ceftolozane-tazobactam (Fig. 2). Regardless, 91.9% of isolates of carbapenem-nonsusceptible Enterobacteriaceae that were concurrently nonsusceptible to ceftazidime-avibactam retained a cefiderocol MIC of ≤4 μg/ml compared with 96.7% of isolates of carbapenem-nonsusceptible Enterobacteriaceae concurrently nonsusceptible to ceftolozane-tazobactam (Fig. 2). Cefiderocol also inhibited 97.8% (222/227) of carbapenem-nonsusceptible Enterobacteriaceae that were concurrently colistin resistant at a cefiderocol MIC of ≤4 μg/ml. The 31 isolates of Enterobacteriaceae with cefiderocol MICs of 8 to 32 μg/ml were 15 isolates of Enterobacter cloacae, 12 isolates of K. pneumoniae, 3 isolates of Enterobacter aerogenes, and 1 isolate of Citrobacter freundii (data not shown). Ceftazidime-avibactam, ceftolozane-tazobactam, and cefepime each demonstrated MIC_90_ values of >64 μg/ml against the same three sets of isolates of carbapenem-nonsusceptible Enterobacteriaceae (Tables 1 and 2).

**TABLE 1:** *In vitro* activities of cefiderocol and comparative agents against 1,022 clinical isolates of carbapenem-nonsusceptible Enterobacteriaceae

Organism(s) (no. of isolates)	Antimicrobial agent	MIC (μg/ml)^*a*^			MIC interpretation (% of isolates)^*b*^		
		Range	MIC_50_	MIC_90_	Susceptible	Intermediate	Resistant
Enterobacteriaceae (1,022)	Cefiderocol	0.004 to 32	1	4			
	Cefepime	≤0.06 to >64	>64	>64	2.8	7.0	90.2
	Ceftazidime-avibactam	≤0.06 to >64	2	>64	77.0	0	23.0
	Ceftolozane-tazobactam	0.25 to >64	>64	>64	1.7	2.0	96.4
	Ciprofloxacin	≤0.12 to >8	>8	>8	11.5	4.2	84.3
	Colistin	≤0.25 to >8	0.5	>8	77.8	0	22.2
	Meropenem	2 to >64	16	>64	0	7.1	92.9
K. pneumoniae (689)	Cefiderocol	0.004 to 32	1	4			
	Cefepime	0.5 to >64	>64	>64	1.2	4.1	94.8
	Ceftazidime-avibactam	≤0.06 to >64	2	>64	86.9	0	13.1
	Ceftolozane-tazobactam	0.5 to >64	>64	>64	0.7	0.7	98.6
	Ciprofloxacin	≤0.12 to >8	>8	>8	5.5	1.3	93.2
	Colistin	≤0.25 to >8	0.5	>8	75.0	0	25.0
	Meropenem	2 to >64	32	>64	0	4.5	95.5
Enterobacter spp. (158)^*c*^	Cefiderocol	0.06 to 32	2	8			
	Cefepime	≤0.06 to >64	64	>64	4.4	10.8	84.8
	Ceftazidime-avibactam	0.12 to >64	>64	>64	37.3	0	62.7
	Ceftolozane-tazobactam	0.25 to >64	>64	>64	3.8	2.5	93.7
	Ciprofloxacin	≤0.12 to >8	>8	>8	21.5	10.1	68.4
	Colistin	≤0.25 to >8	0.5	2	92.4	0	7.6
	Meropenem	2 to >64	8	64	0	11.4	88.6
E. coli (73)	Cefiderocol	0.015 to 4	1	2			
	Cefepime	4 to >64	64	>64	0	11.0	89.0
	Ceftazidime-avibactam	0.12 to >64	0.5	>64	78.1	0	21.9
	Ceftolozane-tazobactam	4 to >64	64	>64	0	4.1	95.9
	Ciprofloxacin	≤0.12 to >8	>8	>8	8.2	4.1	87.7
	Colistin	≤0.25 to >8	0.5	1	95.9	0	4.1
	Meropenem	2 to >64	8	32	0	19.2	80.8
S. marcescens (39)	Cefiderocol	0.015 to 4	0.5	2			
	Cefepime	≤0.06 to >64	16	>64	15.4	18.0	66.7
	Ceftazidime-avibactam	0.12 to >64	1	>64	74.4	0	25.6
	Ceftolozane-tazobactam	0.5 to >64	32	>64	12.8	10.3	76.9
	Ciprofloxacin	≤0.12 to >8	2	>8	46.2	18.0	35.9
	Colistin	8 to >8	>8	>8	0	0	100
	Meropenem	2 to >64	16	>64	0	2.6	97.4
Citrobacter spp. (32)^*d*^	Cefiderocol	0.015 to 8	0.5	2			
	Cefepime	1 to >64	32	>64	18.8	9.4	71.9
	Ceftazidime-avibactam	≤0.06 to >64	2	>64	65.6	0	34.4
	Ceftolozane-tazobactam	4 to >64	>64	>64	0	3.1	96.9
	Ciprofloxacin	≤0.12 to >8	4	>8	25.0	9.4	65.6
	Colistin	≤0.25 to 1	0.5	1	100	0	0
	Meropenem	2 to 64	4	16	0	21.9	78.1
K. oxytoca (31)	Cefiderocol	0.03 to 4	0.25	1			
	Cefepime	1 to >64	16	>64	6.5	25.8	67.7
	Ceftazidime-avibactam	0.12 to >64	1	>64	71.0	0	29.0
	Ceftolozane-tazobactam	2 to >64	32	>64	3.2	9.7	87.1
	Ciprofloxacin	≤0.12 to >8	2	>8	41.9	16.1	41.9
	Colistin	≤0.25 to >8	0.5	1	96.8	0	3.2
	Meropenem	2 to 64	8	32	0	6.5	93.6

a MIC_50_ and MIC_90_ values for an individual genus or species were calculated when >30 isolates were tested. Species of Enterobacteriaceae with <30 isolates were grouped together as genus data.

b Blank spaces indicate that CLSI, EUCAST, and FDA MIC breakpoints were not available for the agent.

c The 158 isolates of Enterobacter spp. were comprised of 137 Enterobacter cloacae, 13 Enterobacter aerogenes, 5 Enterobacter kobei, and 3 Enterobacter asburiae isolates.

d The 32 isolates of Citrobacter spp. were comprised of 28 Citrobacter freundii, 3 Citrobacter koseri, and 1 Citrobacter amalonaticus isolates.

**TABLE 2:** *In vitro* activities of cefiderocol and comparative agents against clinical isolates of carbapenem-nonsusceptible Enterobacteriaceae that demonstrated concurrent nonsusceptibility to ceftolozane-tazobactam or ceftazidime-avibactam

Antimicrobial susceptibility phenotype (no. of isolates)	Antimicrobial agent	MIC (μg/ml)			MIC interpretation (% of isolates)^*a*^		
		Range	MIC_50_	MIC_90_	Susceptible	Intermediate	Resistant
Carbapenem nonsusceptible and nonsusceptible to ceftolozane-tazobactam (1,005)	Cefiderocol	0.004 to 32	1	4			
	Cefepime	0.5 to >64	>64	>64	1.8	6.7	91.5
	Ceftazidime-avibactam	≤0.06 to >64	2	>64	76.6	0	23.4
	Ceftolozane-tazobactam	4 to >64	>64	>64	0	2.0	98.0
	Ciprofloxacin	≤0.12 to >8	>8	>8	10.4	4.2	85.5
	Colistin	≤0.25 to >8	0.5	>8	78.2	0	21.8
	Meropenem	2 to >64	16	>64	0	6.9	93.1
Carbapenem nonsusceptible and nonsusceptible to ceftazidime-avibactam (235)	Cefiderocol	0.03 to 32	2	4			
	Cefepime	0.5 to >64	>64	>64	1.3	3.0	95.7
	Ceftazidime-avibactam	16 to >64	>64	>64	0	0	100
	Ceftolozane-tazobactam	32 to >64	>64	>64	0	0	100
	Ciprofloxacin	≤0.12 to >8	>8	>8	15.7	6.8	77.5
	Colistin	≤0.25 to >8	0.5	>8	83.8	0	16.2
	Meropenem	2 to 64	32	32	0	3.8	96.2

a Blank spaces indicate that CLSI, EUCAST, and FDA MIC breakpoints were not available for the agent.

**FIG 3:**
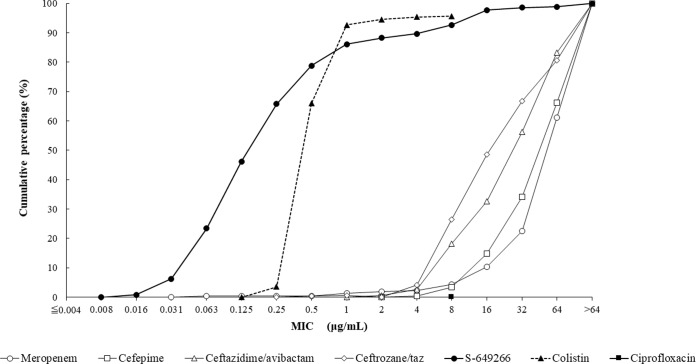
Cumulative cefiderocol MIC distribution (percentage of isolates) for 368 isolates of MDR A. baumannii.

The MIC_50_ and MIC_90_ values for cefiderocol against MDR A. baumannii were 0.25 and 8 μg/ml (Table 3); 89.7% (330/368) of isolates exhibited cefiderocol MICs of ≤4 μg/ml. Colistin (MIC_90_, 1 μg/ml) was the only other agent tested that demonstrated significant *in vitro* activity against isolates of MDR A. baumannii. All colistin-resistant isolates of A. baumannii (*n* = 20) had cefiderocol MICs of ≤4 μg/ml. The cumulative percentage of isolates of MDR A. baumannii inhibited at various MICs of each agent tested is shown in Fig. 3.

**TABLE 3:** *In vitro* activity of cefiderocol and comparative agents against MDR A. baumannii, MDR P. aeruginosa, S. maltophilia, and B. cepacia

Antimicrobial susceptibility phenotype and/or organism(s) (no. of isolates)	Antimicrobial agent	MIC (μg/ml)^*a*^			MIC interpretation (% of isolates)^*b*^		
		Range	MIC_50_	MIC_90_	Susceptible	Intermediate	Resistant
MDR A. baumannii (368)	Cefiderocol	0.015 to >256	0.25	8			
	Cefepime	4 to >64	64	>64	3.3	11.7	85.1
	Ceftazidime-avibactam	≤0.06 to >64	32	>64			
	Ceftolozane-tazobactam	0.5 to >64	32	>64			
	Ciprofloxacin	>8	>8	>8	0	0	100
	Colistin	≤0.25 to >8	0.5	1	94.6	0	5.4
	Meropenem	≤0.06 to >64	64	>64	1.9	0.3	97.8
MDR P. aeruginosa (262)	Cefiderocol	≤0.002 to 32	0.25	1			
	Cefepime	1 to >64	32	>64	13.7	28.2	58.0
	Ceftazidime-avibactam	0.5 to >64	32	>64	36.3	0	63.7
	Ceftolozane-tazobactam	0.5 to >64	>64	>64	24.1	4.6	71.4
	Ciprofloxacin	1 to >8	>8	>8	1.2	5.0	93.9
	Colistin	≤0.25 to 8	1	1	99.6	0	0.4
	Meropenem	≤0.06 to >64	32	>64	3.8	4.2	92.0
S. maltophilia (217)	Cefiderocol	0.004 to 2	0.06	0.25			
	Cefepime	0.25 to >64	32	64			
	Ceftazidime-avibactam	0.25 to >64	8	64			
	Ceftolozane-tazobactam	0.25 to >64	8	>64			
	Ciprofloxacin	1 to >8	2	>8			
	Colistin	≤0.25 to >8	2	>8			
	Meropenem^*c*^	0.12 to >64	>64	>64			
B. cepacia (4)	Cefiderocol	0.004 to 8					
	Cefepime^*c*^	16 to 64					
	Ceftazidime-avibactam	2 to 8					
	Ceftolozane-tazobactam	1 to 4					
	Ciprofloxacin	1 to 4					
	Colistin^*c*^	≤0.25 to >8					
	Meropenem	2 to 4			100	0	0

a MIC_50_ and MIC_90_ values were calculated when >30 isolates were tested.

b Blank species indicate that CLSI, EUCAST, and FDA MIC breakpoints were not available for the agent.

c Pathogen is intrinsically resistant to this antimicrobial agent (5).

**FIG 4:**
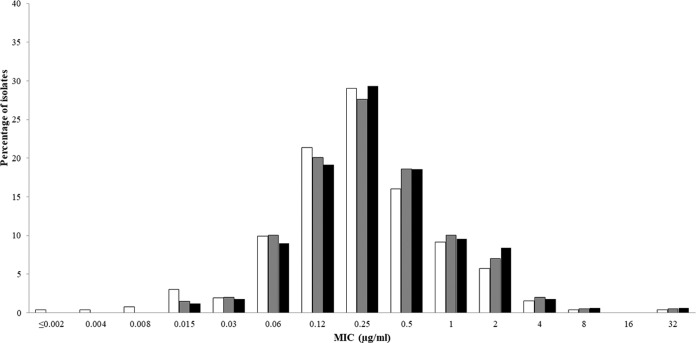
Cefiderocol MIC distributions for all isolates of MDR P. aeruginosa (white bars; *n* = 262), isolates of MDR P. aeruginosa that were concurrently nonsusceptible to ceftolozane-tazobactam (gray bars; *n* = 199), and isolates of MDR P. aeruginosa that were concurrently nonsusceptible to ceftazidime-avibactam (black bars; *n* = 167).

**FIG 5:**
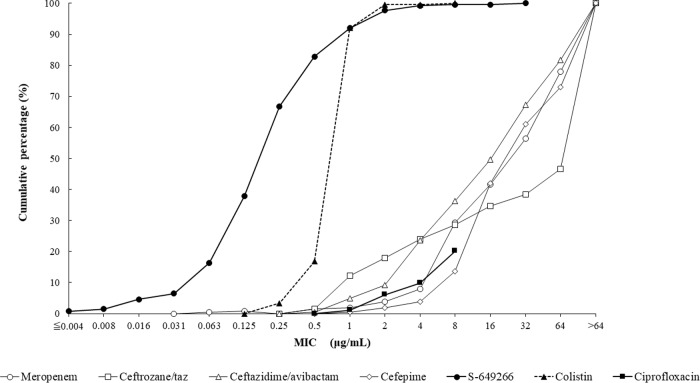
Cumulative cefiderocol MIC distribution (percentage of isolates) for 262 isolates of MDR P. aeruginosa.

The MIC_50_ and MIC_90_ values for cefiderocol against MDR P. aeruginosa were 0.25 and 1 μg/ml, respectively (Table 3). Ceftazidime-avibactam, ceftolozane-tazobactam, and cefepime each demonstrated MIC_90_ values of >64 μg/ml against the same set of isolates of MDR P. aeruginosa. The MIC_50_ and MIC_90_ values for cefiderocol tested against isolates of P. aeruginosa with concurrent MDR and ceftolozane-tazobactam-nonsusceptible phenotypes (*n* = 199) and MDR and ceftazidime-avibactam-nonsusceptible phenotypes (*n* = 167) were 0.25 and 2 μg/ml, respectively, for both sets of isolates (Table 4). A total of 260 (99.2%) isolates of MDR P. aeruginosa exhibited cefiderocol MIC values of ≤4 μg/ml, including 99.0% of ceftolozane-tazobactam-nonsusceptible isolates and 98.8% of ceftazidime-avibactam-nonsusceptible isolates. Cefiderocol MIC distributions for all MDR P. aeruginosa, ceftolozane-tazobactam-nonsusceptible MDR P. aeruginosa, and ceftazidime-avibactam-nonsusceptible MDR P. aeruginosa isolates were very similar (Fig. 4). The cumulative percentage of isolates of MDR P. aeruginosa inhibited at various MICs of each agent tested is shown in Fig. 5.

**TABLE 4:** *In vitro* activity of cefiderocol and comparative agents against MDR P. aeruginosa that demonstrated concurrent nonsusceptibility to ceftolozane-tazobactam or ceftazidime-avibactam

Antimicrobial susceptibility phenotype (no. of isolates)	Antimicrobial agent	MIC (μg/ml)			MIC interpretation (% of isolates)^*a*^		
		Range	MIC_50_	MIC_90_	Susceptible	Intermediate	Resistant
MDR and nonsusceptible to ceftolozane-tazobactam (199)	Cefiderocol	0.015 to 32	0.25	2			
	Cefepime	1 to >64	32	>64	6.0	27.1	66.8
	Ceftazidime-avibactam	1 to >64	32	>64	16.6	0	83.4
	Ceftolozane-tazobactam	8 to >64	>64	>64	0	6.0	94.0
	Ciprofloxacin	1 to >8	>8	>8	0.5	4.5	95.0
	Colistin	≤0.25 to 2	1	1	100	0	0
	Meropenem	0.12 to >64	64	>64	1.5	2.5	96.0
MDR and nonsusceptible to ceftazidime-avibactam (167)	Cefiderocol	0.015 to 32	0.25	2			
	Cefepime	8 to >64	64	>64	1.8	27.5	70.7
	Ceftazidime-avibactam	16 to >64	64	>64	0	0	100
	Ceftolozane-tazobactam	4 to >64	>64	>64	0.6	1.8	97.6
	Ciprofloxacin	1 to >8	>8	>8	0.6	3.0	96.4
	Colistin	≤0.25 to 2	1	1	100	0	0
	Meropenem	4 to >64	64	>64	0	3.0	97.0

a Blank spaces indicate that CLSI, EUCAST, and FDA MIC breakpoints were not available for the agent.

**FIG 6:**
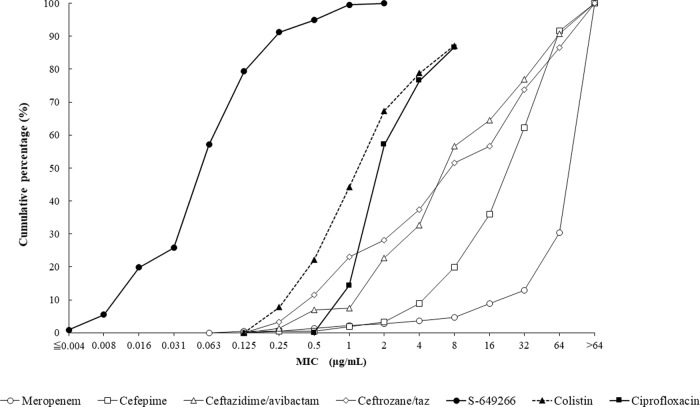
Cumulative cefiderocol MIC distribution (percentage of isolates) for 217 isolates of S. maltophilia.

The MIC_50_ and MIC_90_ values for cefiderocol against S. maltophilia were 0.06 and 0.25 μg/ml, respectively. All S. maltophilia isolates tested had cefiderocol MIC values of ≤2 μg/ml. The MIC_90_s for cefepime, ceftazidime-avibactam, ceftolozane-tazobactam, and meropenem were ≥64 μg/ml, and they were >8 μg/ml for colistin and ciprofloxacin. There are no published CLSI MIC breakpoints for S. maltophilia for any of the antimicrobial agents tested in this study. The cumulative percentage of isolates of S. maltophilia inhibited at various MICs of each agent tested is shown in Fig. 6.

The MICs of cefiderocol for the four isolates of B. cepacia tested in this study were 0.004, 0.008, 0.015, and 8 μg/ml. Too few isolates were collected to generate MIC_50_ and MIC_90_ values.

If the entire data set is considered (*n* = 1,873 isolates) and if species intrinsically resistant to colistin (B. cepacia and Serratia spp.) and species for which colistin MIC breakpoints are not available (S. maltophilia) are excluded (*n* = 260), colistin nonsusceptibility was observed for 13.0% (209/1,613) of isolates tested (173 Klebsiella sp. isolates, 20 A. baumannii isolates, 12 Enterobacter sp. isolates, 3 E. coli isolates, and 1 P. aeruginosa isolate). The cefiderocol MIC range, MIC_50_, and MIC_90_ for colistin-nonsusceptible isolates were 0.03 to 32, 1, and 4 μg/ml; 96.7% (202/209) of colistin-nonsusceptible isolates had cefiderocol MICs of ≤4 μg/ml.

## DISCUSSION

The current study demonstrated cefiderocol to be a more potent antimicrobial agent *in vitro* than cefepime, ceftazidime-avibactam, ceftolozane-tazobactam, ciprofloxacin, and colistin against a recent worldwide collection of clinical isolates of carbapenem-nonsusceptible Enterobacteriaceae, MDR A. baumannii, MDR P. aeruginosa, S. maltophilia, and B. cepacia (Tables 1 and 3). Cefiderocol maintained its potency against isolates of Gram-negative bacilli resistant to colistin and the β-lactam/β-lactamase inhibitor combinations ceftazidime-avibactam and ceftolozane-tazobactam (Tables 2 and 4). Based on MIC_90_s, cefiderocol (MIC_90_, 4 μg/ml) was >16 times more potent than cefepime, ceftazidime-avibactam, and ceftolozane-tazobactam against carbapenem-nonsusceptible isolates of Enterobacteriaceae. Against MDR A. baumannii, cefiderocol (MIC_90_, 8 μg/ml) was >8 times more potent than cefepime, ceftazidime-avibactam, and ceftolozane-tazobactam; only colistin (MIC_90_, 1 μg/ml) exhibited more potent activity than cefiderocol against MDR A. baumannii. Cefiderocol exhibited an MIC_90_ (1 μg/ml) against MDR P. aeruginosa that was >64 times more potent than that for cefepime, ceftazidime-avibactam, and ceftolozane-tazobactam and similar to that for colistin (MIC_90_, 1 μg/ml). Cefiderocol demonstrated potent activity against S. maltophilia (MIC_90_, 0.25 μg/ml) while all six comparative agents were inactive.

Cefiderocol at a concentration of ≤4 μg/ml inhibited all isolates (217/217) of S. maltophilia, 99.2% (260/262) of isolates of MDR P. aeruginosa, 97.0% (991/1,022) of isolates of carbapenem-nonsusceptible Enterobacteriaceae, and 89.7% (330/368) of isolates of MDR A. baumannii. The highest MIC observed for cefiderocol for both carbapenem-nonsusceptible Enterobacteriaceae and MDR P. aeruginosa was 32 μg/ml. Against MDR A. baumannii, 38 isolates (10.3% of isolates) had cefiderocol MICs of >4 μg/ml, with 256 μg/ml being the highest MIC observed. One isolate of B. cepacia of the four isolates tested had a cefiderocol MIC of 8 μg/ml; all other isolates had cefiderocol MICs of ≤0.015 μg/ml.

A limited number of previous studies have determined the *in vitro* activity of cefiderocol against surveillance study isolates of Gram-negative bacilli as well as against Gram-negative bacilli with molecularly characterized ESBLs and carbapenemases and isolates resistant to carbapenems by mechanisms other than carbapenemases (13–15, 25–29). In a recent study, Falagas and colleagues tested cefiderocol by broth microdilution, using ID-CAMHB prepared according to the approved CLSI protocol, against a collection of 471 carbapenem-resistant isolates of Enterobacteriaceae, P. aeruginosa, and A. baumannii collected from inpatients in Greek hospitals (26). They reported MIC_90_s for cefiderocol ranging from 0.5 to 1 μg/ml for individual species of Enterobacteriaceae and MIC_90_s of 0.5 μg/ml for both P. aeruginosa and A. baumannii. In another recent study, in which cefiderocol was also tested by the broth microdilution method using ID-CAMHB prepared according to the approved CLSI protocol, a 2014–2015 collection of clinical isolates of Gram-negative bacilli from North America and Europe was tested (27). In that study, MICs of cefiderocol were ≤4 μg/ml for 99.9% of all Enterobacteriaceae (MIC_90_, 0.5 to 1 μg/ml), for 97.0% of meropenem-nonsusceptible (MIC, ≥2 μg/ml) Enterobacteriaceae (MIC_90_, 1 to 4 μg/ml), for 99.9% of all P. aeruginosa isolates (MIC_90_, 0.5 μg/ml), for 100% (353/353) of meropenem-nonsusceptible (MIC, ≥4 μg/ml) P. aeruginosa isolates (MIC_90_, 0.5 μg/ml), for 97.6% of all A. baumannii isolates (MIC_90_, 1 μg/ml), for 96.9% of meropenem-nonsusceptible (MIC, ≥4 μg/ml) A. baumannii isolates (MIC_90_, 1 μg/ml), for 100% of isolates of S. maltophilia (MIC_90_, 0.25 to 0.5 μg/ml), and for 93.8% of B. cepacia isolates (27). Slight geographic differences in susceptibilities to cefiderocol were identified in this study, with isolates from Europe demonstrating cefiderocol MIC_90_s that were one doubling dilution higher than those of isolates from North America for all Enterobacteriaceae (1 versus 0.5 μg/ml) and meropenem-nonsusceptible P. aeruginosa (1 versus 0.5 1 μg/ml) and two doubling dilutions higher for meropenem-nonsusceptible Enterobacteriaceae (4 versus 1 μg/ml) (27). The cefiderocol MIC_90_ was one doubling dilution higher for North American isolates of S. maltophilia than for isolates from Europe (0.5 versus 0.25 μg/ml) (27).

Other studies have reported the *in vitro* activity of cefiderocol against isolates of Gram-negative bacilli harboring molecularly defined mechanisms of resistance. One of these studies reported that isolates of Enterobacteriaceae harboring ESBLs (e.g., CTX-type, SHV-type, and TEM-type), KPC-type carbapenemases, VIM-type and IMP-type carbapenemases, and OXA-type carbapenemases all had cefiderocol MICs of ≤4 μg/ml as did 90% (44/49) of NDM-1-positive isolates (13). However, more recent studies from the United Kingdom (28) and France (29) using broth medium prepared with apotransferrin, a less potent and reliable iron chelator than Chelex, have reported that cefiderocol was less active than reported by Kohira et al. (13) against some isolates of Gram-negative bacilli carrying NDM-positive, OXA-type, and KPC carbapenemases. Another study reported MICs of cefiderocol of ≤2 μg/ml for clinical isolates of P. aeruginosa (*n* = 33) positive for GIM-1, IMP-type, or SPM-1 carbapenemases and that 87.5% (14/16) of VIM-positive isolates of P. aeruginosa had cefiderocol MICs of ≤4 μg/ml (15). The same study also reported that IMP-1-, OXA-51-, and OXA-58-positive isolates of A. baumannii (*n* = 29) had cefiderocol MICs of ≤4 μg/ml while selected isolates harboring OXA-23 or OXA-24 were less susceptible to cefiderocol (15). Yamano et al. reported that no significant changes in the *in vitro* activity of cefiderocol were observed in isolates of P. aeruginosa with OprD deficiency or overproduction of efflux pumps or in isolates of K. pneumoniae with OmpK deficiency (30) while another study has reported cefiderocol MICs of ≥8 μg/ml associated with certain isolates of Enterobacteriaceae demonstrating porin loss (28). A weakness in the current study was that molecular analysis of the isolates included was not performed to correlate cefiderocol MICs to genetic markers, and, therefore, the current results cannot be compared directly with other isolate data sets containing molecularly characterized isolates.

Cefiderocol is a promising, novel siderophore cephalosporin currently in clinical development and represents a potentially significant advance in the treatment options available to clinicians to care for patients infected with antimicrobial-resistant Gram-negative bacilli. The intent of the current study was to add to the limited amount of available *in vitro* data in which cefiderocol MICs were determined against Enterobacteriaceae and nonfermentative Gram-negative bacilli using the recently approved CLSI method for producing ID-CAMHB (17–19). Our testing demonstrated that cefiderocol possesses potent *in vitro* activity against carbapenem-nonsusceptible Enterobacteriaceae, MDR A. baumannii, MDR P. aeruginosa, S. maltophilia, and B. cepacia. Cefiderocol MICs were ≤4 μg/ml for 96.2% (1,801/1,873) of all isolates of carbapenem-nonsusceptible and MDR Gram-negative bacilli tested in the current study. The potent *in vitro* activity of cefiderocol was maintained against both ceftazidime-avibactam-nonsusceptible and ceftolozane-tazobactam-nonsusceptible isolates of Gram-negative bacilli, as well as against isolates nonsusceptible to colistin.

## MATERIALS AND METHODS

### Bacterial isolates.

Isolates of Gram-negative bacilli tested in this study (*n* = 1,873) were selected from the International Health Management Associates, Inc. ([IHMA] Schaumburg, IL), 2014–2016 surveillance study frozen stock culture collection based on their known antimicrobial susceptibility testing phenotypes and/or their species identification; 413 isolates were selected from 2014, 1,123 isolates were from 2015, and 337 isolates were from 2016. Isolates of Enterobacteriaceae (*n* = 1,022) were chosen based on their carbapenem-nonsusceptible phenotype (meropenem MIC of ≥2 μg/ml) (5). Isolates of A. baumannii (*n* = 368) and P. aeruginosa (*n* = 262) were chosen because they demonstrated an amikacin-resistant (MIC, ≥32 μg/ml), ciprofloxacin-resistant (MIC, ≥4 μg/ml), and meropenem-resistant (MIC, ≥16 μg/ml) MDR phenotypes (5, 31). Isolates of S. maltophilia (*n* = 217) and B. cepacia (*n* = 4) were chosen based solely on their identities and the preponderance of each of these species to demonstrate MDR phenotypes. All isolates were originally grown from specimens of patients with a documented intra-abdominal, urinary tract, skin and soft tissue, lower respiratory tract, or bloodstream infection. Isolates tested in this study were limited to one per patient. The identities of all isolates were confirmed by IHMA using matrix-assisted laser desorption ionization–time of flight (MALDI-TOF) mass spectrometry (Bruker Daltonics, Billerica, MA). The 1,873 isolates of Gram-negative bacilli were collected by medical center laboratories in 52 countries. Specifically, 995 isolates were collected by medical laboratories in 24 countries in Europe, 399 isolates were from 10 countries in Latin America, 220 isolates were from 2 countries in North America, 155 isolates were from 8 countries in Asia, 61 isolates were from 3 countries in the South Pacific, 29 isolates were from 2 countries in Africa, and 14 isolates were from 3 countries in the Middle East.

### Antimicrobial susceptibility testing.

CLSI standard methods were employed to generate broth microdilution panels as well as to perform panel inoculation, incubation, reading, and interpretation (5, 24). All aspects of antimicrobial susceptibility testing were performed on-site at IHMA. Broth microdilution panels included the following antimicrobial agents: cefiderocol (doubling dilution range tested, 0.002 to 256 μg/ml), cefepime (0.06 to 64 μg/ml), ceftazidime-avibactam (0.06/4 to 64/4 μg/ml), ceftolozane-tazobactam (0.06/8 to 64/8 μg/ml), ciprofloxacin (0.12 to 8 μg/ml), colistin (0.25 to 8 μg/ml), and meropenem (0.06 to 64 μg/ml). Cefiderocol and ceftolozane were obtained from Shionogi & Co., Ltd. (Osaka, Japan). Avibactam was obtained from Biochempartner (Wuhan, China). All other antimicrobial agents were purchased from the U.S. Pharmacopeia (Rockville, MD). Cefiderocol was dissolved and diluted in sterile normal saline (17). BBL cation-adjusted Mueller-Hinton broth (CAMHB) (Becton-Dickinson, Sparks, MD) was used for all antimicrobial susceptibility testing and was prepared according to the manufacturer's instructions (5, 24). Cefiderocol was tested using iron-depleted CAMHB (ID-CAMHB) that was prepared by adding 100 g of Chelex 100 resin (Bio-Rad Laboratories, Hercules, CA) to 1 liter of autoclaved CAMHB, and the suspension was stirred for 2 h at room temperature (23°C) to remove cations in the medium. The iron-depleted broth was then filtered using a 0.2-μm-pore-size filter to remove the resin, and its pH was adjusted to 7.3 using 0.1 M hydrochloric acid. The ID-CAMHB was then supplemented with calcium (CaCl_2_), magnesium (MgCl_2_), and zinc (ZnSO_4_) to final concentrations of 22.5 μg/ml (range, 20 to 25 μg/ml), 11.25 μg/ml (range, 10 to 12.5 μg/ml), and 10 μM (0.56 μg/ml; range 0.5 to 1.0 μg/ml), respectively, and again passed through a 0.2-μm-pore-size filter. The method of preparation of ID-CAMHB described above was approved by the CLSI Subcommittee on Antimicrobial Susceptibility Testing (17–19, 32) and has supplanted previous medium preparation methods, including those using 20 μM human apotransferrin, because of MIC reproducibility issues, using Iso-Sensitest broth because it has only a single manufacturer (13–15, 25, 32). The final concentration of iron in ID-CAMHB prepared using the above method is ≤0.03 μg/ml (17).

The broth microdilution panels included growth control wells for both CAMHB and ID-CAMHB. The panels were incubated at 35°C for 20 h in ambient air before MIC endpoints were read. ID-CAMHB did not significantly affect the growth of any quality control or test organism. Reading the MIC of cefiderocol was contingent on the presence of strong growth in the ID-CAMHB growth control (i.e., a button of approximately 2 mm or greater). The cefiderocol MIC was read as the first panel well in which isolate growth was significantly reduced (i.e., a button of <1 mm or light/faint turbidity) relative to the growth observed in the ID-CAMHB growth control well. The method described here for reading MIC endpoints for cefiderocol was approved by the CLSI Subcommittee on Antimicrobial Susceptibility Testing but has not yet been published (17–19, 32).

Cefiderocol currently does not have approved MIC interpretative breakpoints. In this study, cefiderocol MICs were analyzed by determining the numbers (percentages) of isolates with MICs of ≤4 μg/ml. A concentration of cefiderocol of ≤4 μg/ml was used to group isolates because *in vitro* pharmacokinetic/pharmacodynamic and animal infection models that recreate human drug exposure have demonstrated that cefiderocol possesses bactericidal killing and clinical efficacy against isolates of Enterobacteriaceae, P. aeruginosa, and A. baumannii with cefiderocol MICs of 4 μg/ml (33–35). Katsube and coworkers reported that the proposed human dose of cefiderocol of 2 g every 8 h, using 3-h infusions, maintained the free-drug concentration of cefiderocol in plasma above 4 μg/ml for at least 75% of the dosing interval in patients with normal kidney function, resulting in >90% probability of target attainment and, therefore, probable clinical success (33). CLSI interpretive criteria, when available (5), and FDA interpretive criteria for ceftazidime-avibactam (36) were used to interpret MICs of the comparator agents tested. For colistin, CLSI interpretive criteria were used to interpret MICs for P. aeruginosa and A. baumannii. Colistin lacks CLSI or FDA breakpoints for Enterobacteriaceae; therefore, the European Committee on Antimicrobial Susceptibility Testing (EUCAST) MIC breakpoints for Enterobacteriaceae were applied to Enterobacteriaceae tested against colistin (susceptible, ≤2 μg/ml; resistant, ≥4 μg/ml) (37).

Quality control testing was performed each day of testing using E. coli ATCC 25922, P. aeruginosa ATCC 27853, and K. pneumoniae ATCC 700603. All quality control results were within specified CLSI ranges (5) including CLSI approved, but not yet published, ranges for cefiderocol (E. coli ATCC 25922, 0.06 to 0.5 μg/ml; P. aeruginosa ATCC 27853, 0.06 to 0.5 μg/ml) (17–19, 32).
